# Gallic Acid in Menorrhagia

**Published:** 1843-11

**Authors:** 


*Gallic Acid in Menorrhagia.*—Professor Simpson stated at a meeting of the *Med. Chirurg. Soc. of Edinburgh*, April 19th, that for the last year he had employed gallic acid in some cases of menorrhagia, with the most successful results. Like all other remedies directed against that disease, it had also occasionally failed in his hands. Some of the cases which had completely yielded under its use were of an old standing, and aggravated description. He gave it during the intervals, as well as during the discharge, in doses of from ten to twenty grains per day made into pills. It had this advantage over most other anti-hemorrhagic medicines, that it had no constipating effect upon the bowels. He was first induced to use it from finding a case of very obstinate menorrhagia get well under the use of Ruspini's styptic, after many other remedies had utterly failed, and from it being alleged that gallic acid was the active ingredient in that styptic. He suggested whether the anti-hemorrhagic properties of some of our common astringent drugs may not depend upon the gallic acid as much or more than upon the tannin which they contain, or upon the tannin becoming changed into gallic acid within the body.—*Amer. Journ. of the Med. Sci.*, from *Lond. and Edin. Monthly Journ. Med. Sci.*, July, 1843.

